# An Analysis of Male Breast Cancer Incidence, Mortality, and Disease Burden Among Commonwealth Countries

**DOI:** 10.7759/cureus.85608

**Published:** 2025-06-09

**Authors:** Nuha Amri, Sandeep Babu Bhaskaran Pillai

**Affiliations:** 1 Department of Surgery, United Lincolnshire Hospitals NHS Trust, Lincoln, GBR

**Keywords:** cancer epidemiology, commonwealth regions, disease burden, health inequities, income disparities, male breast cancer, public health analysis

## Abstract

Introduction and aim

Male breast cancer is a rare disease, and it remains inadequately researched globally. Due to its rarity and lack of widespread awareness, this disease often presents in an advanced stage, leading to higher mortality rates compared to the more common female breast cancer. Increasing age, the presence of genetic mutations, such as BRCA2, and exposure to certain hormonal and environmental factors have been cited as factors increasing the risk of this disease. This study aimed to investigate the incidence, death, and burden of male breast cancer, measured in years lived with disability (YLD) across the following three socioeconomic categories in the Commonwealth of Nations: high-, middle-, and low-income.

Methods

This study uses a retrospective cohort design to analyze data from the Global Burden of Disease (GBD) database from 1991 to 2021 to identify long-term trends and disparities. Descriptive statistics summarize breast cancer outcomes, while Analysis of Variance (ANOVA) and Tukey’s honestly significant difference (HSD) tests identify significant differences across the socioeconomic categories.

Findings

Significant differences in male breast cancer incidence, mortality, and YLDs were found across the different socioeconomic categories. Middle-income Commonwealth regions had the highest incidence and mortality rates, whereas high-income regions had the lowest. These outcomes highlight the potential role of socioeconomic factors, wherein residents in high-income regions benefit from efficient early detection programs and advanced healthcare treatments, and policies.

Conclusion

In lower- and middle-class regions, there is a pressing need to improve the healthcare system and increase cancer awareness. The worldwide occurrence of advanced male breast cancer can be reduced by addressing these discrepancies through focused efforts to aid early detection, thereby improving prognoses. This study contributes to the limited body of literature regarding male breast cancer by highlighting the significance of socioeconomic factors on breast cancer outcomes and the need for equitable healthcare access.

## Introduction

Background

Male breast cancer is a relatively rare condition, accounting for less than 1% of all breast cancer cases globally. The World Health Organization (WHO) estimates that between 0.5% and 1% of cases of breast cancer affect the male population, which translates to approximately 2,800 new cases annually in the United States alone and more than 38,000 new cases globally in the years 2020 and 2021 [1]. Approximately 2.3 million new cases were reported globally in 2022 [2]. Although breast cancer is primarily considered a female disease, with one in eight women at risk of developing breast cancer in her lifetime, men also face a lifetime risk, estimated at about one in 833 [3]. Furthermore, male breast cancer frequently manifests at a later stage, thereby leading to a high mortality rate. According to the American Cancer Society, an estimated 530 males in the United States will die of breast cancer in 2024.

Key risk factors underlying the development of male breast cancer include age, family history, genetic mutations, therapeutic radiation exposure, and high estrogen levels. Deleterious mutations in the BRCA2 genes, Klinefelter’s syndrome, undescended testicles, injuries to the testis, and exposure to atomic radiation, as in nuclear holocaust survivors, have also been causally linked to an increase in incidence of this disease [4-6].

Due to the sparse incidence of male breast cancer, the currently available data are almost entirely derived from small cohort series. Rarity also leads to gross under-reporting of this disease, as many countries do not have a national database of male breast cancers. A low index of suspicion from the affected individual and their primary treating doctor, caused by lack of awareness, is also a factor in the delay in detection and timely referral. The incidence is more frequent in Blacks than in non-Hispanic Whites in the United States, which is in contrast to the higher incidence of breast cancer in White females [2].

Initial evaluation entails recording a thorough history, including the use of medications, such as estrogenic steroids, alcohol and tobacco use, exposure to therapeutic or accidental radiation, family history, clinical examination, diagnostic mammography, ultrasound scan of the breast and axillary lymph nodes, and core needle biopsy with or without radiologic guidance. Ninety percent of male breast cancer cases are found to be estrogen receptor-positive and HER2-positive, in comparison to merely 20% HER2 positivity in females. Curative management options such as surgery for the breast and axilla, radiotherapy, and systemic adjuvant treatment modalities are highly similar to those used in the more common female breast cancer types [7].

Moreover, it should be noted that male breast cancer patients are seldom represented in clinical trials and, consequently, most of the current evidence in managing this disease is extrapolated from the results of trials involving females. Gaining knowledge regarding the incidence pattern, treatment outcomes, prognosis, and mortality figures in male breast cancer would aid in creating gender-specific early diagnostic tools, implementing prevention and treatment plans that could increase survival rates, and lowering overall mortality [7,8].

Commonwealth regions

This study focuses on analyzing male breast cancer incidence, mortality, and disease burden across Commonwealth regions categorized by their economic status as follows: high-income, middle-income, and low-income. Because of their shared history and diverse geographical factors, ethnicity, and economic backgrounds, the Commonwealth regions provide a unique paradigm to analyze how socioeconomic factors could influence incidence, mortality, and treatment-related disability outcomes. High-income Commonwealth regions include Antigua and Barbuda, Australia, the Bahamas, Barbados, Brunei Darussalam, Canada, Cyprus, Malta, New Zealand, Saint Kitts and Nevis, Seychelles, Singapore, Trinidad and Tobago, and the United Kingdom.

Middle-income Commonwealth regions include Belize, Botswana, Cameroon, Dominica, Eswatini, Fiji, Ghana, Grenada, Guyana, India, Jamaica, Kiribati, Lesotho, Malaysia, the Maldives, Mauritius, Namibia, Nauru, Nigeria, Pakistan, Papua New Guinea, Saint Lucia, Saint Vincent and the Grenadines, Samoa, Solomon Islands, South Africa, Sri Lanka, Tonga, Tuvalu, Vanuatu, and Zambia.

Low-income Commonwealth regions include Bangladesh, Kenya, Malawi, Mozambique, Rwanda, Sierra Leone, Uganda, and the United Republic of Tanzania. These regions differ significantly in terms of healthcare systems, access to care, and other socioeconomic factors, all of which could affect male breast cancer outcomes. The classification of these countries within the Global Burden of Disease (GBD) database is predicated on the Socio-Demographic Index (SDI). The SDI is a composite metric that takes into account factors such as fertility rate, education level, and income per capita [9]. An examination of these regions is expected to facilitate a comprehensive analysis of how different socioeconomic and healthcare profiles may affect male breast cancer outcomes.

Existing literature

Research has indicated that male breast cancer tends to be more common in high-income countries, likely because these countries have better healthcare systems and advanced diagnostic and reporting tools [10]. Quite surprisingly, the mortality rates are also greater in high-income Commonwealth countries despite the facilities for early diagnosis and effective treatment, but the difference in mortality is less pronounced than the difference in incidence. Survival rates tend to be lower for male breast cancer in general than for female breast cancer, and this is attributed to a lack of awareness and delays in diagnosis [11]. In addition to death rates, the burden of this disease also includes the impact on the quality of life and economic costs, with high-income regions encountering significant health expenses and lost productivity.

However, there is limited data from low-income and middle-income Commonwealth nations, and this creates a knowledge gap. As most of the available studies focus on high-income countries, and data on populations living in less affluent regions are significantly lacking, there is a need to factor in these differences while considering resource allocation for early detection of the disease and installation of advanced treatment units. For example, studies have highlighted the role of socioeconomic disparities in breast cancer outcomes, emphasizing the need for improved access to care in low- and middle-income regions, among other needs [12,13].

Linking income to male breast cancer risk

As a socioeconomic measure, income level plays a significant role in analyzing health outcomes, including the incidence and mortality of cancer. Low incidence and mortality rates are a result of improved treatment choices, early detection programs, and access to top-quality healthcare services in high-income areas. Conversely, challenges encountered in middle- and low-income areas include restricted access to healthcare, delayed diagnosis, and inadequate treatment choices, all of which may lead to elevated rates of disease burden and mortality.

This study explores how these income-related disparities manifest in the context of male breast cancer, particularly focusing on how income categories influence the availability and effectiveness of healthcare interventions, which, in turn, affect incidence, mortality, and years lived with disability (YLD). By examining data from the GBD database over a three-decade period, this study aimed to uncover the underlying socioeconomic determinants that contribute to these disparities and provide evidence to inform targeted public health strategies.

Research question and hypothesis

This study aimed to address the following research question: “Is there a significant difference in the incidence, mortality, and disease burden of male breast cancer between the three Commonwealth regions?” The null hypothesis (H0) proposes that there is no impact of socioeconomic status on the incidence, mortality, or disease burden of male breast cancer, indicating no significant differences across regions. The alternative hypothesis (H1) suggests that there are significant disparities among low-, middle-, and high-income Commonwealth regions. These differences could be attributed to variations in healthcare access, quality of medical facilities, public health initiatives, genetic factors, lifestyle choices, and the prevalence of risk factors such as obesity and alcohol consumption that could disrupt hormonal balance and cause an increase in estrogen levels, thereby raising the risk of getting cancer. Geographic variations, cultural and dietary practices, the influence of tobacco use, and even genetic diversity could influence the overall differences in disease rates among these countries.

Contribution to published evidence

This study aimed to enhance the understanding of male breast cancer by analyzing its incidence, mortality, and disease burden across various Commonwealth regions. By differentially interpreting the data from countries according to varying income levels, this study seeks to provide insights that are often missing in global research. Additional statistical tests are conducted to provide detailed insights into the risk and survival rates for male breast cancer. By focusing on the particular challenges and opportunities within these regions, the study intends to draw attention to the necessity of focused public health interventions and policies. This research also emphasizes the importance of awareness and education about male breast cancer, potentially influencing policy and healthcare practices to improve early diagnosis and treatment. The overall goal of this study is to bridge the knowledge gap and improve the understanding and management of male breast cancer, which ultimately will lower the disease’s burden and raise the survival rates of men who are afflicted with it.

## Materials and methods

Study design

This study investigates the GBD database for trends and variations in breast cancer outcomes over a three-decade period (1991-2021) among the various socioeconomic groups in the Commonwealth regions using a retrospective cohort approach. While the longitudinal nature of the dataset aligns with a retrospective approach, the analysis is ecological in design, relying on aggregated, population-level data. The main goal is to identify and analyze differences in the incidence, mortality, and disability burden of breast cancer in low-, middle-, and high-income areas; the disability burden is measured in YLDs. As it allows for the analysis of existing data to identify patterns and correlations over a prolonged period, the retrospective cohort design is deemed appropriate.

Data sources

Data for this study were acquired from the GBD database. This database provides comprehensive estimates of the disease burden, including absolute case counts for incidence, mortality, and YLDs for various medical conditions, including breast cancer. The dataset encompasses the three decades of data, offering a robust foundation for examination of long-term trends and disparities. The GBD database was selected because of its wide scope and standardized data compilation, which ensures reasonably accurate and consistent data across geographical boundaries. No age-standardization or data imputation was performed.

Setting

This study was focused on Commonwealth regions that were categorized by income levels (low, middle, and high). This wide geographical and economic scope renders it possible to analyze breast cancer outcomes in detail in a variety of socioeconomic contexts. This study intends to investigate how different healthcare access levels and socioeconomic situations affect male breast cancer outcomes by examining these specific locations.

Time frame

Data from January 1, 1991, to December 31, 2021, were examined in this study. This 30-year time frame enables the assessment of long-term trends and the impact of various health policies and interventions over time. The extended time frame also facilitates the tracking of any notable changes in male breast cancer incidence, mortality, and disease burden that could be attributed to advancements in medical technology for the detection or treatment of male breast cancer or any major shifts in public health initiatives and strategies.

Inclusion and exclusion criteria

The inclusion criteria for this study required the availability of data on breast cancer incidence, mortality, and YLDs from the GBD database. Furthermore, the information was categorized by economic levels within the Commonwealth regions, specifically, high-, middle-, and low-income countries. By contrast, the exclusion criteria omitted data points from countries where breast cancer outcomes were incomplete or missing. Additionally, regions that were not classified within the Commonwealth or that did not have an income categorization were excluded from the analysis.

Study variables

The primary outcome variables for this study include breast cancer incidence, mortality, and YLDs. These outcomes were evaluated in relation to various independent variables, including income categories (low, middle, and high) within the Commonwealth regions and the specific years when the data were captured.

Statistical methods

Descriptive Analysis

Descriptive statistics such as mean and standard deviation were calculated to summarize breast cancer outcomes (incidence, mortality, and YLDs) within each income category of the Commonwealth regions. These statistics provided an overview of the distribution and variability of breast cancer outcomes over the study period. Visual aids, such as graphs and charts, were used to clearly illustrate these differences.

Comparative Analysis

The comparative analysis involved the use of Analysis of Variance (ANOVA) to assess disparities in breast cancer outcomes across different income categories within Commonwealth regions over the three decades that were studied. Post hoc tests were performed following ANOVA to identify specific group differences by comparing the means of each subgroup with the means of the other groups. Tukey’s honestly significant difference (HSD) test was used for this comparison.

Approvals and Registrations

This project has adhered to the relevant institutional guidelines and regulatory standards set forth by the University of Edinburgh. Since the study involves a retrospective cohort analysis using publicly available country-level routine data sources, it does not require further ethical approval. However, it is understood that any submission for publication in research journals may necessitate additional ethical review.

## Results

Descriptive analysis

Incidence

**Figure 1:**
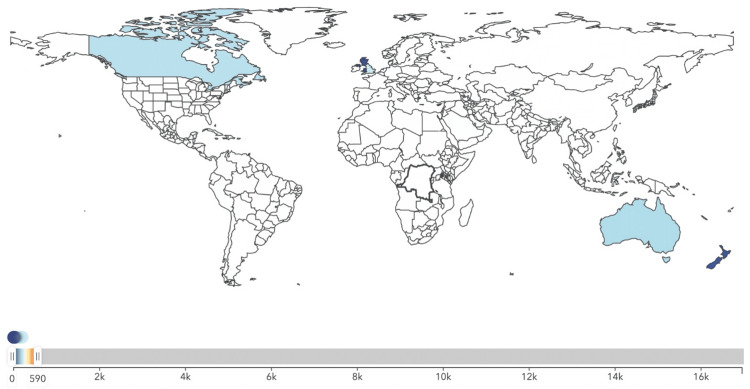
Incidence of male breast cancer in high-income Commonwealth regions in the year 2021.

**Figure 2:**
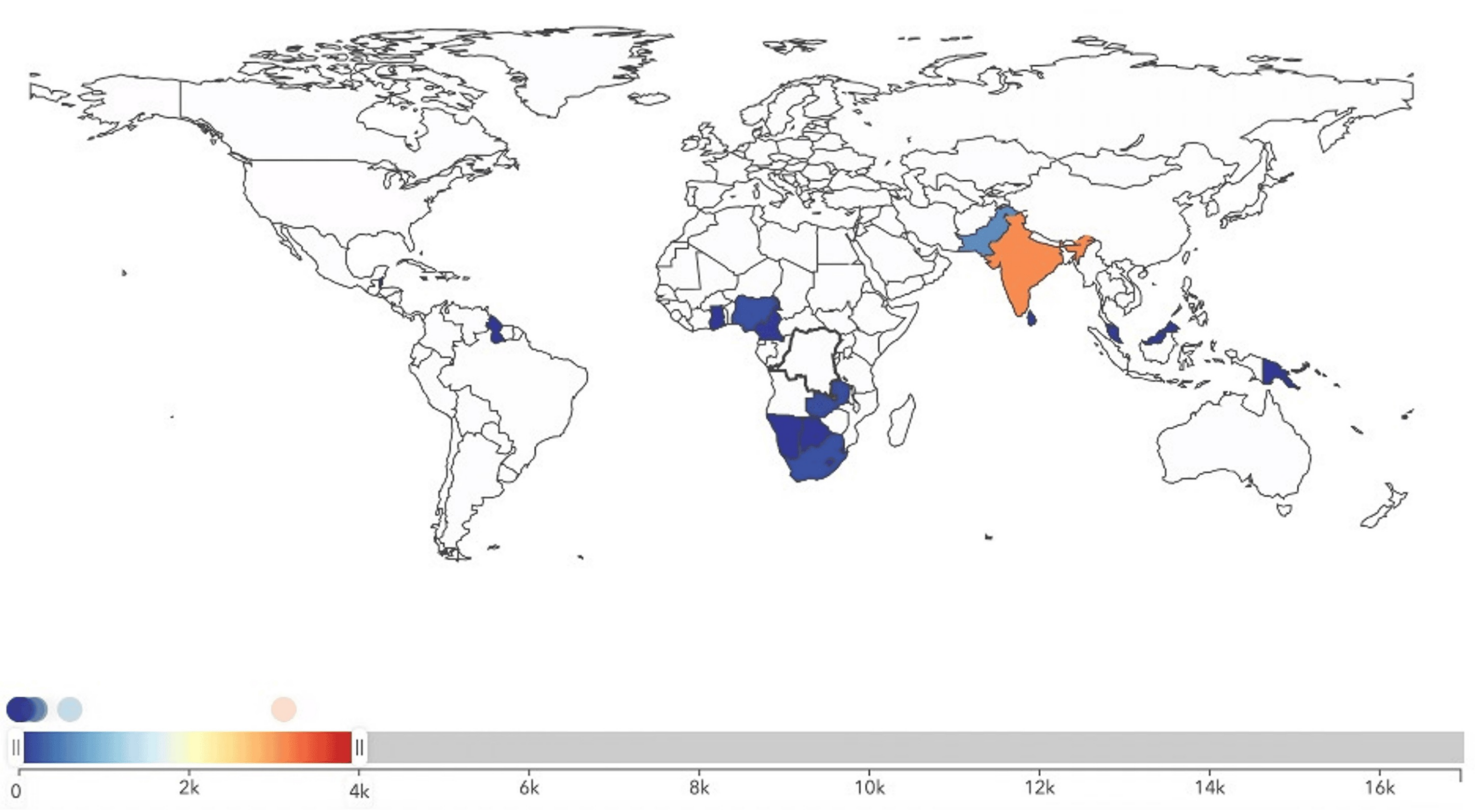
Incidence of male breast cancer in middle-income Commonwealth regions in the year 2021.

**Figure 3:**
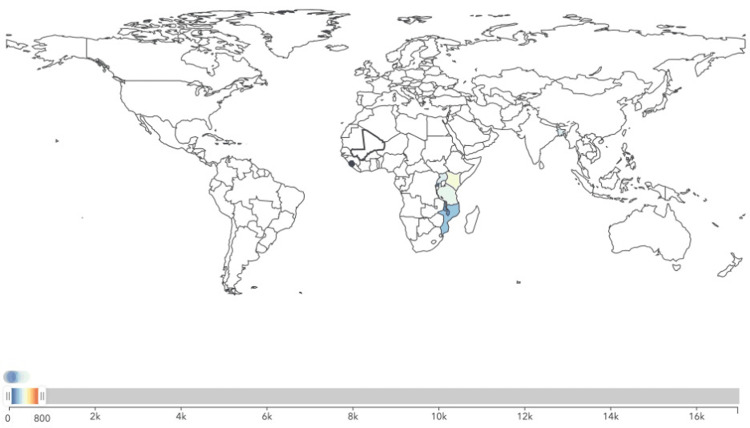
Incidence of male breast cancer in low-income Commonwealth regions in the year 2021.

The analysis revealed significant disparities in male breast cancer incidence across the different income categories. The mean incidence rate was highest in middle-income regions (2456.91) and lowest in high-income regions (532.50) (Figures 1-3). The standard deviation was also highest in middle-income regions (1021.68), indicating greater variability in incidence rates.

Deaths

For deaths due to male breast cancer, middle-income regions had the highest mean death rate (1718.14), and high-income regions had the lowest (172.25) mean death rate. The variability was again highest in middle-income regions (536.84).

YLDs

**Figure 4:**
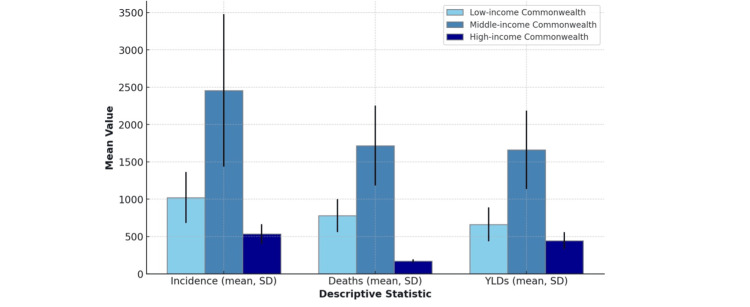
Mean incidence, deaths, and years lived with disability (YLD) with standard deviations across commonwealth income categories. This figure illustrates the mean values of incidence, deaths, and years lived with disability (YLD) across different Commonwealth income categories (low, middle, and high), with standard deviations as error bars.

YLDs due to male breast cancer followed a similar pattern, with middle-income regions having the largest mean YLDs (1661.84) and high-income regions exhibiting the lowest (444.78). The standard deviation was also the greatest in middle-income regions (524.99). The findings from the descriptive analysis are summarized in Table 1 and are visually represented in Figure 4.

**Table 1:** Descriptive statistics for incidence, deaths, and YLDs. YLD: years lived with disability

Descriptive statistic	Low-income Commonwealth	Middle-income Commonwealth	High-income Commonwealth
Incidence (mean, SD)	1021.77 (341.97)	2456.91 (1021.68)	532.50 (133.42)
Deaths (mean, SD)	778.82 (221.94)	1718.14 (536.84)	172.25 (22.80)
YLDs (mean, SD)	662.27 (227.56)	1661.84 (524.99)	444.78 (112.27)

Comparative analysis

Incidence

ANOVA results: The ANOVA test revealed a significant F-statistic of 92.54, with a p-value of approximately 1.58×10^-20^. This indicates that there are significant differences in male breast cancer incidence rates among the different income categories within Commonwealth regions.

Post hoc analysis: To determine which specific groups differ from each other, a Tukey’s HSD test was performed. The results show that high-income regions have significantly lower incidence rates compared to both low-income (mean difference: -489.27, p<0.001) and middle-income regions (mean difference: -1924.41, p<0.001). Additionally, low-income regions have significantly lower incidence rates compared to middle-income regions (mean difference: -1435.14, p<0.001).

Deaths

ANOVA results: The ANOVA for death rates exhibited a significant F-statistic of 85.23, with a p-value of approximately 2.35×10^-19^. This suggests significant disparities in death rates due to male breast cancer across the varying income categories.

Post hoc analysis: The Tukey’s HSD test provided the following results: the mean difference in death rates between high-income and low-income regions was -606.57 (p<0.001), indicating that high-income regions have significantly lower death rates compared to low-income regions. Between high-income and middle-income regions, the mean difference was -1545.89 (p<0.001), showing that high-income regions also have significantly lower death rates compared to middle-income regions. Lastly, the mean difference between low-income and middle-income regions was -939.31 (p<0.001), suggesting that low-income regions have significantly lower death rates than middle-income regions.

Years Lived With Disability

ANOVA results: The ANOVA test for YLDs revealed significant differences among the income categories, with a p<0.001 and an F-statistic of 78.15. This indicates that the differences in YLDs across the income categories are statistically significant.

Post hoc analysis: The Tukey’s HSD test provided the following results: the mean difference in YLDs between high-income and low-income regions is -217.49 (p<0.001), indicating that high-income regions have significantly lower YLDs compared to low-income regions. For high-income versus middle-income regions, the mean difference is -1217.06 (p<0.001), demonstrating that high-income regions also have significantly lower YLDs than middle-income regions. Finally, the mean difference between low-income and middle-income regions is -999.57 (p<0.001), suggesting that low-income regions have significantly lower YLDs compared to middle-income regions.

In summary, these results demonstrate that high-income regions consistently exhibit a significantly lower rate of male breast cancer incidence, deaths, and YLDs compared to low- and middle-income regions. Middle-income regions tend to have the highest rates across all three parameters, likely due to various socioeconomic and healthcare-related factors. A summary of these results is provided in Tables 2, 3.

**Table 2:** ANOVA results for incidence, deaths, and years living with disability.

Metric	F-statistic	p-Value
Incidence	92.54	1.58×10^-20^
Deaths	85.23	2.35×10^-^^19^
YLDs	78.15	3.17×10^-^^18^

**Table 3:** Tukey’s honestly significant difference test results for incidence, deaths, and years living with disability. YLD: years living with disability

Comparison	Incidence mean difference	Incidence p-value	Deaths mean difference	Deaths p-value	YLDs mean difference	YLDs p-value
High-income versus low-income	-489.27	<0.001	-606.57	<0.001	-217.49	<0.001
High-income versus middle-income	-1924.41	<0.001	-1545.89	<0.001	-1217.06	<0.001
Low-income versus middle-income	-1435.14	<0.001	-939.31	<0.001	-999.57	<0.001

## Discussion

Findings and context

The analysis highlights significant disparities in male breast cancer incidence, deaths, and YLDs across different income categories within the Commonwealth regions. High-income Commonwealth regions consistently exhibited lower rates across all three disease parameters compared to low- and middle-income regions. These findings are consistent with existing literature indicating that socioeconomic factors play a crucial role in breast cancer epidemiology [12,13]. Studies have demonstrated that improved healthcare infrastructure, early detection initiatives, and advancements in treatment strategies have proved advantageous to high-income regions in reducing incidence, mortality, and disease burden [14]. Moreover, high-income Commonwealth countries with well-established cancer registries facilitate precise data collection and subsequent effective public health initiatives [15].

Geographic and genetic factors

The geographic distribution of Commonwealth countries could influence the incidence of male breast cancer due to varying levels of ultraviolet radiation exposure, dietary habits, and environmental factors. For example, Australia, despite being a high-income Commonwealth country, exhibits low incidence rates, which could be attributed to successful public health campaigns and preventative measures [16,17]. On the contrary, middle-income regions such as India and Nigeria have higher incidence rates, which could be attributed to less effective healthcare systems and late-stage diagnoses [18,19]. Furthermore, the larger number of total male breast cancer cases in middle-income Commonwealth regions may be partly due to their larger populations. This population base can result in a greater number of total cases, despite individual incidence rates being elevated in high-income Commonwealth countries.

Lawson and Glenn reported evidence linking a number of malignancies to chronic viral infections, such as those caused by the human papillomavirus and Epstein-Barr virus [20]. However, the exact correlation and/or causation between these infections and breast cancer remains unclear. Although viral infections can exacerbate the clinical picture, most agree that the increased incidence of infectious illnesses and the lack of awareness of these illnesses in the low- and middle-income commonwealth regions worsen the late presentation of cancer patients [20].

Moreover, genetic predisposition plays a significant role in the incidence of breast cancer. Studies have indicated that residents of Canada and the United States have a higher prevalence of BRCA mutations, which increases the risk of breast cancer [21]. Conversely, the lower incidence rates observed in some high-income regions might be influenced by effective genetic counseling and preventive measures that mitigate the impact of genetic predisposition. Additionally, genetic diversity in middle-income countries could contribute to varying breast cancer risks, potentially leading to higher incidence rates in these regions [22].

Environmental and occupational factors

Environmental and workplace exposures can significantly affect the risks of various cancers. Although direct evidence linking pesticide exposure to male breast cancer is limited, there is evidence revealing a connection between pesticide exposure and female breast cancer. For example, studies have demonstrated that harmful chemicals found in industrial environments and organophosphate insecticides are associated with an increased risk of breast cancer in women [23,24]. It is possible that similar mechanisms could affect men, but, due to the different genetic and hormonal makeup of men, these effects might remain hidden until the disease has progressed to an advanced stage. This suggests opportunities for new research to explore the correlation in the male population. Consequently, to reduce these hazards, robust workplace regulations must be implemented, and occupational safety standards must be raised. Policies aimed at minimizing carcinogen exposure and ensuring regular health monitoring can help protect vulnerable populations, thereby lowering cancer incidence rates.

Limitations and future directions

This study is limited to Commonwealth countries, which represent a subset of the global population. Considering that male breast cancer constitutes barely 1% of all breast cancer cases around the world, the observed disparities and complexities highlighted in this study underscore the significant impact of this disease. These findings underline the magnitude of complexity and variation in epidemiology that is present in more prevalent diseases, including breast cancer in females. To ensure a comprehensive understanding of male breast cancer epidemiology, future studies should include a heterogeneous global population. This would help identify broad trends and factors that influence breast cancer incidence and outcomes and provide deep insights into the complexities of more prevalent diseases.

Furthermore, this study does not take into consideration inequalities across socioeconomic categories, such as those between urban and rural areas, which may have a substantial impact on access to healthcare and the outcomes [25,26]. While the GBD dataset offers broad, standardized coverage, it relies on modeled estimates that may be affected by underreporting or missing data in some countries, particularly in low- and middle-income regions. Future studies could also investigate the impact of healthcare policies, cultural beliefs, and environmental factors that might contribute to the observed discrepancies [13,25].

Global collaboration and knowledge sharing

A key to better diagnosis and treatment of male breast cancer could be an increase in international collaboration and exchange of knowledge among nations of varying financial levels. Initiatives such as that launched by the Union for International Cancer Control demonstrate how partnerships can effectively aid in the development of appropriate treatment guidelines given available resources [27]. Expansion or creation of similar collaborations could strengthen healthcare systems in low-income regions, thereby enhancing patient outcomes.

Awareness and prevention

High-income Commonwealth countries have likely seen lower incidence rates of male breast cancer due to raised awareness and preventive measures. Effective public health campaigns, regular screening programs, and advanced healthcare services in these regions play a crucial role in the early detection and prevention of this disease. To minimize disparities in breast cancer outcomes, similar strategies need to be reinforced and adopted in low- and middle-income countries. Therefore, reducing male breast cancer prevalence globally would require the implementation of community-based education initiatives and expansion of access to facilities for screening, diagnosis, and treatment, particularly in less affluent regions [28,29].

## Conclusions

In conclusion, the differences seen in the incidence, deaths, and YLDs of male breast cancer underscore the importance of tackling socioeconomic inequities in population education and healthcare access for early detection. The study revealed that middle-income Commonwealth regions had the highest mean incidence (2456.91), mortality (1718.14), and YLDs (1661.84), whereas high-income regions had the lowest mean incidence (532.50), mortality (172.25), and YLDs (444.78). Targeted interventions to improve healthcare infrastructure and accessibility in low-income regions are crucial. Further research with more granular data and consideration of current trends is important for developing effective public health strategies to reduce the burden of male breast cancer across all regions. Policymakers must prioritize cancer control programs and ensure equitable distribution of healthcare resources to mitigate these disparities.
